# The Role of Fatty Acid Metabolism in Drug Tolerance of Mycobacterium tuberculosis

**DOI:** 10.1128/mbio.03559-21

**Published:** 2022-01-11

**Authors:** Camila G. Quinonez, Jae Jin Lee, Juhyeon Lim, Mark Odell, Christopher P. Lawson, Amararachukwu Anyogu, Saki Raheem, Hyungjin Eoh

**Affiliations:** a Department of Molecular Microbiology and Immunology, Keck School of Medicine, University of Southern California, Los Angeles, California, USA; b Department of Life Sciences, Faculty of Science and Technology, University of Westminster, London, United Kingdom; c School of Life Sciences, University of Lincoln, Lincoln, United Kingdom; d Strathclyde Institute of Pharmacy and Biomedical Sciences, University of Strathclyde, Glasgow, United Kingdom; e School of Biomedical Sciences, University of West London, London, United Kingdom; Washington University School of Medicine in St. Louis

**Keywords:** tuberculosis, drug tolerance, metabolomics, methylcitrate cycle, fatty acids, acetate, drug tolerance, tuberculosis

## Abstract

Mycobacterium tuberculosis can cocatabolize a range of carbon sources. Fatty acids are among the carbons available inside the host’s macrophages. Here, we investigated the metabolic changes of the fatty acid-induced dormancy-like state of *M. tuberculosis* and its involvement in the acquisition of drug tolerance. We conducted metabolomics profiling using a phosphoenolpyruvate carboxykinase (PEPCK)-deficient *M. tuberculosis* strain in an acetate-induced dormancy-like state, highlighting an overaccumulation of methylcitrate cycle (MCC) intermediates that correlates with enhanced drug tolerance against isoniazid and bedaquiline. Further metabolomics analyses of two *M. tuberculosis* mutants, an ICL knockdown (KD) strain and PrpD knockout (KO) strain, each lacking an MCC enzyme—isocitrate lyase (ICL) and 2-methylcitrate dehydratase (PrpD), respectively—were conducted after treatment with antibiotics. The ICL KD strain, which lacks the last enzyme of the MCC, showed an overaccumulation of MCC intermediates and a high level of drug tolerance. The PrpD KO strain, however, failed to accumulate MCC intermediates as it lacks the second step of the MCC and showed only a minor level of drug tolerance compared to the ICL KD mutant and its parental strain (CDC1551). Notably, addition of authentic 2-methylisocitrate, an MCC intermediate, improved the *M. tuberculosis* drug tolerance against antibiotics even in glycerol medium. Furthermore, wild-type *M. tuberculosis* displayed levels of drug tolerance when cultured in acetate medium significantly greater than those in glycerol medium. Taken together, the fatty acid-induced dormancy-like state remodels the central carbon metabolism of *M. tuberculosis* that is functionally relevant to acquisition of *M. tuberculosis* drug tolerance.

## INTRODUCTION

Tuberculosis (TB) accounted for ∼1.5 million deaths in 2020, including 214,000 people who were HIV positive (1). This disease is an ancient scourge caused by infection with Mycobacterium tuberculosis, a slow-growing bacterium (2). This opportunistic bacterium can remain viable but phenotypically quiescent for decades before causing active TB (3). It is estimated that 1.7 billion people (around 23% of the world’s population) harbor *M. tuberculosis* bacilli in a dormant state, although this feature is controversial due to suboptimal diagnostic methods to detect latent TB infection (4). An estimated 10 million people develop active TB each year (5). Patients with active TB can be cured by the conventional TB treatment method, but it requires 6 to 9 months with four first-line drugs (isoniazid [INH], rifampin, pyrazinamide, and ethambutol) (1). Moreover, the long treatment duration with a multidrug combination is associated with significant rates of patient noncompliance and a rapid rise of antibiotic-resistant strains, which poses a public health threat (6, 7). Therefore, there is an urgent need to identify new drug targets to improve the aged regimen.

*M. tuberculosis* exhibits metabolic plasticity, including the ability to cocatabolize multiple carbon sources simultaneously (8). This metabolic strategy is often used to adapt to a range of environmental stresses, including nutrient starvation, hypoxia, or antibiotic treatment. A small fraction of *M. tuberculosis* cells enter a reduced-growth state that is relatively insensitive to these environmental stresses until conditions become favorable for them to regrow and become metabolically active (9–16). The ability to switch between replicating and nonreplicating states was reported to happen through rerouting its metabolic fluxes as an adaptive response to its surrounding environmental stresses. Indeed, *M. tuberculosis* in a nonreplicating state is less susceptible to the antimicrobial effects of the environmental stresses (17, 18). This metabolic versatility also determines the susceptibility of *M. tuberculosis* to antibiotics (19), allowing survival even in the presence of lethal doses of bactericidal drugs (12, 13, 15, 20, 21). The cells can survive in such an adverse environment for a prolonged period of time in the absence of resistance as a result of genetic mutations (22). Therefore, understanding the intricate metabolic remodeling that *M. tuberculosis* uses to survive during infection and dormancy is critical in the development of new drugs.

Here, we attempted to uncover the carbon source-dependent metabolic changes of *M. tuberculosis*, especially in the central carbon metabolism that leads to drug tolerance against isoniazid (INH) or bedaquiline (BDQ). To elucidate this, we used three *M. tuberculosis* mutant strains deficient in genes involved in the fatty acid catalytic node within the central carbon metabolism. These rendered *M. tuberculosis* viable but unable to grow in medium containing model fatty acids such as acetate or propionate as the sole carbon source, demonstrating a drug-tolerant phenotype. We applied liquid chromatography mass spectrometry (LC-MS) metabolomics to elucidate the fatty acid-defined, antibiotic-induced *M. tuberculosis* central carbon metabolism remodeling using INH and BDQ, two clinically relevant anti-TB drugs. We revealed that a phosphoenolpyruvate carboxykinase (PEPCK)-deficient mutant (Δ*pckA*) strain undergoes a series of metabolic remodeling cascades arising from the lack of a gluconeogenic carbon flux that enables *M. tuberculosis* to evade bactericidal effects of antibiotics when cultured in fatty acid, not in glycerol, medium. Outcomes of this study point to a correlation between drug tolerance and an overaccumulation of the methylcitrate cycle (MCC) intermediates, which might trigger the global metabolic rearrangements that contribute to improve drug tolerance. Two MCC mutant strains—one lacking the isocitrate lyase 1 gene (*icl1*) and the other lacking the 2-methylcitrate dehydratase gene (*prpD*)—were characterized, and their behavior supports the role of overaccumulation of MCC intermediates in the acquisition of drug tolerance of *M. tuberculosis*.

## RESULTS

Metabolic enzymes in the fatty acid catalytic node of *M. tuberculosis* central carbon metabolism are required for *M. tuberculosis* not only to consume fatty acids as carbon sources *in vitro* but also to survive *in vivo* (23, 24). PEPCK, encoded by *pckA*, catalyzes the first committed step of gluconeogenesis and is the sole enzyme that converts oxaloacetate (OAA) to phosphoenolpyruvate (PEP) (23). PEPCK has been proposed as a potential drug target as the *pckA*-deficient *M. tuberculosis* Δ*pckA* mutant fails to replicate during the acute phase of infection within mice. The Δ*pckA* mutant was rapidly cleared from the infected mouse lungs by day 56, suggesting that *M. tuberculosis* relies on gluconeogenic carbon sources such as fatty acids for its growth *in vivo* and the establishment of infection. In the present study, we showed that when acetate, a model fatty acid, was supplied as the sole carbon source, the Δ*pckA* strain changes its metabolic state to a dormancy-like (nonreplicating) mode. We also observed that the fatty acid-mediated dormancy-like state of the Δ*pckA* strain was associated with an overaccumulation of the MCC intermediates and accompanied metabolic rearrangement. This metabolic remodeling is directly or indirectly related to a high level of drug tolerance.

### TCA cycle and MCC intermediates overaccumulated in the Δ*pckA* strain cultured in acetate medium.

**FIG 1:**
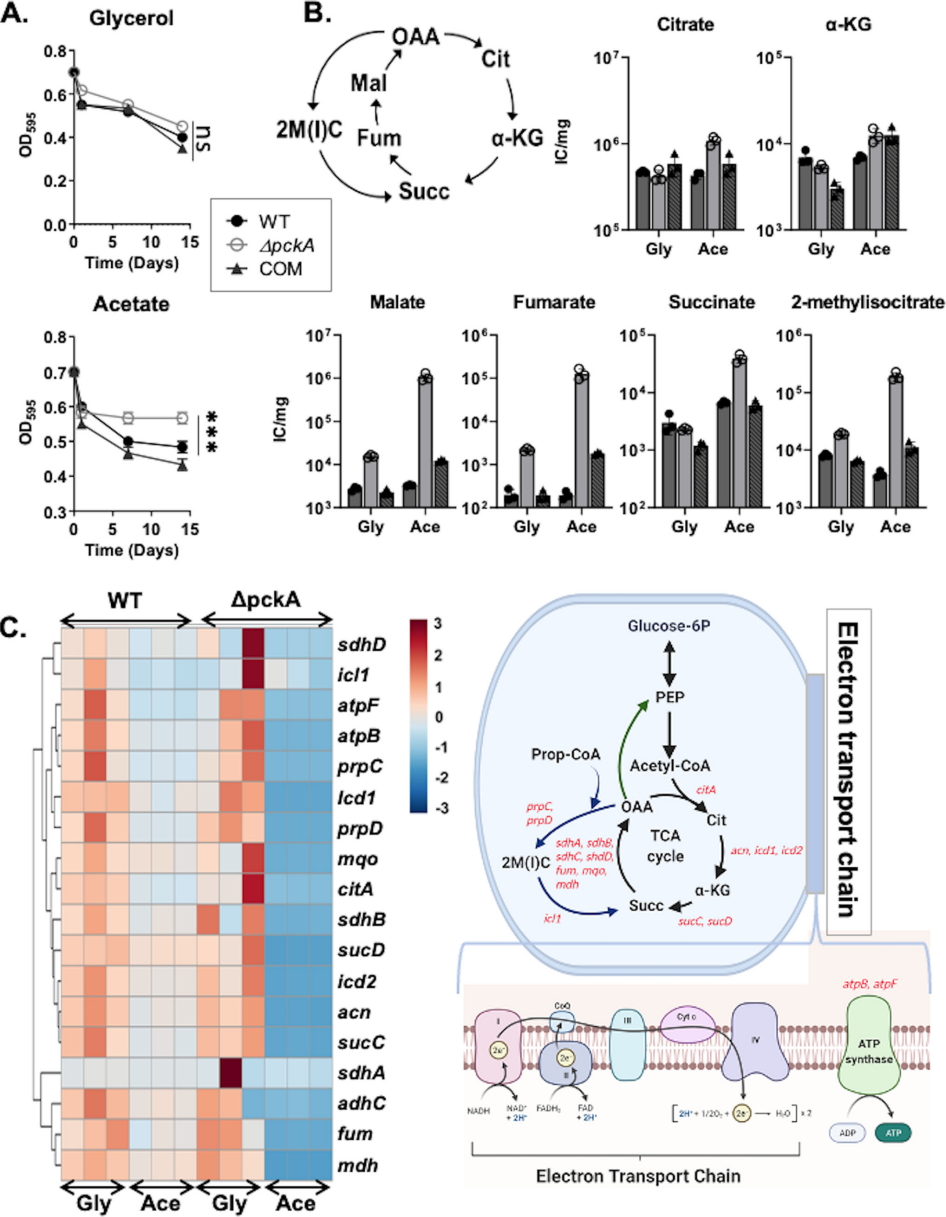
Phenotypic changes in the Δ*pckA* mutant compared to the Erdman wild-type (WT) and complement (COM) strains in carbon-dependent media. (A) Optical density (OD_595_) kinetics were used to quantify the persister formation after treating WT, Δ*pckA*, and COM cells with 100 μg/mL DCS in m7H9 medium containing glycerol (top) and acetate (bottom). All values are the average of experimental triplicates ± standard error of the mean (SEM) and representative of at least two independent experiments. ***, *P* < 0.001, and ns, not significant, by ANOVA. (B). Δ*pckA* mutant-specific remodeling of the TCA cycle and MCC. Shown are intrabacterial pool sizes of intermediates of the TCA cycle and MCC in the three *M. tuberculosis* strains harvested after culturing in glycerol (Gly) or acetate (Ace)-containing medium. Total bar heights indicate the intrabacterial pool sizes depicted by ion counts/mg. (C) Heat map of mRNA expression levels of the TCA cycle, MCC, and ATP synthase genes in the WT or Δ*pckA* strain in glycerol or acetate medium relative to expression of the housekeeping gene, *sigA*. The dark blue to dark red color gradient denotes lower to higher expression determined by comparison with average values of all conditions. Threshold cycle (ΔΔ*C_T_*) values relative to those of glycerol medium were used for the heat map (left panel). A schematic depicting the pathways and genes tested (red font) is shown in the right panel. Green and blue arrows indicated the PEPCK activity and MCC, respectively. Adapted from “Electron Transport Chain,” BioRender.com (2021) (https://app.biorender.com/biorender-templates).

Multiple lines of evidence have shown that PEPCK is essential for *in vitro* growth in medium containing fatty acids but is dispensable for growth when carbohydrates are supplied as carbon sources (23). To confirm these findings, we characterized the *in vitro* growth phenotypes of the *M. tuberculosis* wild-type (WT), a Δ*pckA* mutant, and a *pckA* complemented (COM) strain using Middlebrook 7H9 liquid medium (m7H9) containing either glycerol or a model fatty acid, acetate, as the sole carbon source. The Δ*pckA* mutant failed to increase the biomass when cultured in m7H9 containing acetate (acetate m7H9), but the growth kinetics were almost identical to those of the WT or COM strain in m7H9 containing glycerol (glycerol m7H9) (see Fig. S1A and B in the supplemental material) (23, 25). Monitoring of viable CFU showed that the Δ*pckA* mutant underwent neither net replication nor death in acetate m7H9 (Fig. S1C) (26). The loss of replicating capacity of *M. tuberculosis* without discernible death has been phenotypically associated with an increased level of drug tolerance. We therefore assessed the effect of acetate-induced Δ*pckA* strain nonreplication on drug tolerance by monitoring the optical density (OD) or CFU in the presence of d-cycloserine (DCS), a cell-lysing antibiotic known to generate a drug-tolerant subpopulation, termed persisters (27). As expected, we observed that *M. tuberculosis* with a *pckA* deficiency exhibited an increased persister-forming rate after treatment with a bactericidal dose of DCS in acetate m7H9 compared to that in glycerol m7H9 (Fig. 1A; Fig. S1D and E). When cultured in glycerol m7H9, the Δ*pckA* mutant was initially less susceptible to the DCS effect (at 1 day) compared to that of the WT or COM strain. However, the Δ*pckA* strain gradually succumbed to the effect of DCS and ultimately, the level of sensitivity to DCS became similar to that of the other two strains (at days 7 and 14) (Fig. 1A, upper panel). In contrast, Δ*pckA* strain growth in acetate m7H9 monitored by OD initially revealed a decrease (day 1). However, growth remained static after that, especially during the phase between days 7 and 14, the period known to form the drug-tolerant persisters in response to treatment with DCS (Fig. 1A, lower panel) (27).

10.1128/mbio.03559-21.1 FIG S1 (A to C) *In vitro* growth kinetics of the Erdman (WT), Δ*pckA* mutant, and complement (COM) strains in carbon defined medium containing 0.2% (vol/vol) glycerol (A) or 0.2% (wt/vol) acetate (B). (C) CFU/mL kinetics of each strain in 0.2% (wt/vol) acetate medium for 6 days. (D and E) Killing curve of WT, Δ*pckA*, and COM cells after treatment with 100 μg/mL d-cycloserine (DCS). An exponentially growing population of each strain cultured in 0.2% glycerol (D) or 0.2% (wt/vol) acetate (E) medium was exposed to DCS. Viable counts (CFU/mL) were counted by enumerating the colonies in m7H10 agar medium. All values are the average of independent triplicates ± SEM. ns, not significant; ***, *P* < 0.01 by Student’s unpaired *t* test. Download FIG S1, TIF file, 2.7 MB. Copyright © 2022 Quinonez et al. 2022 Quinonez et al. https://creativecommons.org/licenses/by/4.0/ This content is distributed under the terms of the Creative Commons Attribution 4.0 International license.

To identify the metabolic shifts behind the acetate-induced dormancy-like state and drug tolerance of the Δ*pckA* mutant, we used a filter culture-based metabolomics profiling method (11). A semi-untargeted metabolomics analysis of the WT and Δ*pckA* strains was conducted to identify the metabolites and pathways that were uniquely altered in the Δ*pckA* mutant when cultured in acetate medium (see Fig. S2A to C in the supplemental material). A clustered heat map and principal-component analysis (PCA) indicated that the acetate-induced metabolic alterations of the Δ*pckA* mutant were different from those of the WT in acetate medium or the Δ*pckA* mutant in glycerol medium (Fig. S2A and B). Of 231 metabolites detected, the volcano plot and pathway enrichment analyses pinpointed that upregulated metabolites included the intermediates of the reductive branch of the tricarboxylic acid (TCA) cycle, such as aspartate (a surrogate of OAA), malate, fumarate, and succinate, as well as those of the MCC, such as 2-methylcitrate (2MC) and 2-methylisocitrate (2MI) (collectively 2MIC) (Fig. 1B; Fig S2C and D). The metabolomics and CFU analysis of the WT and Δ*pckA* strains suggested that the defective acetate catabolism of the Δ*pckA* mutant led to a dormancy-like, drug-tolerant state, and this may be associated with the formation of persisters. Targeted metabolomics focusing on the intermediates in the central carbon metabolism showed that *pckA* deficiency caused an overaccumulation of intermediates in both the TCA cycle and MCC (Fig. 1B; Fig. S2D). The accumulation level of the reductive TCA cycle branch intermediates of the Δ*pckA* mutant was by far greater than those of oxidative TCA cycle branch intermediates α-ketoglutarate (α-KG) and citrate: ∼21-fold for aspartate, 17-fold for succinate, 58-fold for fumarate, 69-fold for malate, and 10-fold for the MCC intermediates (2-methylcitrate and 2-methylisocitrate) (Fig. 1B; Fig. S2D).

10.1128/mbio.03559-21.2 FIG S2 Metabolomics analysis of the Erdman wild-type (WT) strain in acetate (A) medium and the Δ*pckA* mutant in glycerol (G) and acetate (A) media. (A) Clustered heat map depicting levels of ∼231 *M. tuberculosis* metabolites of the WT in acetate (A [red]) medium and the Δ*pckA* mutant in glycerol (G [blue]) and acetate (A [green]) media. Data were parsed using uncentered Pearson’s correlation with centroid linkage clustering and rendered using the MetaboAnalyst program (version 4.0). Data are depicted on a log_2_ scale relative to the average of individual metabolite abundance. (B) Three-dimensional principal-component analysis (3D-PCA). Dot colors match the clustered heat map condition bar colors. (C) Volcano plot of upregulated Δ*pckA* metabolites in acetate (A) medium (green box) against upregulated WT metabolites in acetate medium (red box). (D) Targeted metabolomic profiling of WT and Δ*pckA* cells cultured in either glycerol or acetate medium. Shown are the intrabacterial pool sizes of TCA and the methylcitrate cycle (MCC) intermediates of the WT in acetate (A) medium and the *ΔpckA* mutant in both glycerol (G) and acetate (A) media. Cit, citrate, Pyr, pyruvate; α-KG, α-ketoglutarate; Succ, succinate; Fum, fumarate; Mal, malate; 2M(I)C, 2-methyl(iso)citrate; Asp, aspartate (a surrogate of OAA); OAA, oxaloacetate. All values are the average of independent triplicates ± SEM. *, *P* < 0.05, **, *P* < 0.01, and ***, *P* < 0.001, by Student’s unpaired *t* test. Of 231 metabolites analyzed, the metabolisms of the Δ*pckA* mutant in glycerol medium and WT in acetate medium were relatively similar. However, the metabolism of the Δ*pckA* mutant in acetate medium is clearly different from those under the preceding two conditions, indicating a unique metabolic remodeling takes place in the Δ*pckA* mutant in acetate medium resulting in a dormancy-like state. In the volcano plot, it becomes clear that intermediates involved in the reductive TCA cycle branch and the MCC are among the most significant upregulations. Download FIG S2, TIF file, 2.7 MB. Copyright © 2022 Quinonez et al. 2022 Quinonez et al. https://creativecommons.org/licenses/by/4.0/ This content is distributed under the terms of the Creative Commons Attribution 4.0 International license.

### PEPCK deficiency causes metabolic slowdown of TCA cycle activity in acetate medium.

To examine whether the accumulation of reductive TCA cycle branch intermediates of the Δ*pckA* mutant in acetate m7H9 was attributable to the catalytic slowdown arising from the PEPCK deficiency-mediated obstruction of gluconeogenic carbon flux, we first monitored the mRNA levels of genes encoding the TCA cycle and MCC enzymes. The quantitative reverse transcription-PCR (qRT-PCR) results indicated that mRNA levels of Δ*pckA* TCA cycle and MCC genes were globally downregulated in acetate m7H9 compared to those in glycerol m7H9. In comparison, the expression levels of TCA cycle genes in the WT were almost identical between the two carbon conditions (Fig. 1C, left panel). The genes examined included those encoding MCC enzymes (*prpC*, *prpD*, *acn*, and *icl1*), those encoding the reductive TCA cycle branch enzymes (*mdh*, *mqo*, *fum*, *sucC* and -*D*, and *sdhA* to -*D*), those encoding oxidative TCA cycle branch enzymes (*citA*, *acn*, *icd1*, and *icd2*), and those encoding ATP synthases (*atpB* and *atpF*) (Fig. 1C, right panel). A downregulated expression of *prpC*, *acn*, and *prpD*, the genes encoding the first three steps of MCC with minor downregulation of *icl1* expression, may explain the overaccumulation of the MCC intermediates. The targeted metabolomics profile of the Δ*pckA* mutant in acetate m7H9 explained that overaccumulation of the TCA cycle and MCC intermediates could be attributed to the obstructed gluconeogenic carbon flux (Fig. 1B). The acetate-induced slowdown of the Δ*pckA* mutant TCA cycle activity was further examined by the ^13^C labeling patterns of TCA cycle intermediates. We monitored TCA cycle intermediates by transferring the WT, Δ*pckA*, or COM strain to Middlebrook 7H10 agar medium (m7H10) containing uniformly ^13^C-labeled acetate ([U-^13^C]acetate), and the metabolome was sampled after a 24-h incubation. Normal TCA cycle activity results in the progressive assimilation of acetate-based C_2_ units, manifest by the accumulation of higher-order ^13^C_2_-based isotopologues when *M. tuberculosis* was cultured in [U-^13^C]acetate m7H10. The ^13^C labeling patterns showed the downshift of ^13^C-labeled α-KG, malate, and succinate isotopologues of the Δ*pckA* mutant from M + 4 (+4 ^13^C labeled) to M + 2 (+2 ^13^C labeled), revealing acetate-induced slowdown of TCA cycle activity in the Δ*pckA* mutant compared to the activity of the WT. Intriguingly, acetate-induced slowdown of TCA cycle activity in the COM strain did not fully restore to WT levels (see Fig. S3 in the supplemental material). The lack of a complete restoration in the COM strain suggests that the complementation was not fully analogous to the WT metabolic state, despite the use of the native promoter to drive the complementary *pckA* gene. ^13^C isotopologue analysis and qRT-PCR results confirmed that the acetate-induced metabolic remodeling of the Δ*pckA* mutant primarily arose from a catalytic slowdown, rather than an induction of the TCA cycle and MCC activities. We thought that the acetate-induced slowdown of Δ*pckA* TCA cycle activity would be associated with a decreased NADH/NAD ratio as the TCA cycle serves as the main NADH source. Unexpectedly, we detected an increased NADH/NAD ratio in the Δ*pckA* mutant compared to that of the WT under acetate growth conditions (see Fig. S4A and B in the supplemental material). This may be attributable to downregulation of the electron transport chain (ETC), an activity required to recycle NAD from NADH. Indeed, we found that the NAD level in the Δ*pckA* mutant was significantly lower than that of the WT, compared to the depletion rates of NADH, when acetate was provided as the single carbon source (Fig. S4C and D).

10.1128/mbio.03559-21.3 FIG S3 Isotopologue profile of WT, Δ*pckA*, and COM TCA cycle intermediates. The three strains were cultured in [U-^13^C]acetate medium for 24 h. Individual ^13^C isotopologues of TCA cycle intermediates were expressed as the percentage relative to the total abundance of all ion species (labeled and unlabeled) corresponding to the metabolite of interest. The isotopologue distribution of TCA cycle intermediates, except for monoisotopic ^12^C mass (M + 0), is depicted. M + 1, singly ^13^C labelled; M + 2, doubly ^13^C labeled, and so on. Black asterisks indicate ^13^C_2_-labeled isotope species (indicative of incorporation of a single acetate unit), whereas gray circles indicate ^13^C_4_-labeled species (indicative of accumulation of multiple acetate units arising from canonical TCA cycle activity). All values were average of independent experimental triplicates ± SEM. Cit, citrate; α-KG, α-ketoglutarate; Succ, succinate; Mal, malate; OAA, oxaloacetate; MCC, methylcitrate cycle. Download FIG S3, TIF file, 2.7 MB. Copyright © 2022 Quinonez et al. 2022 Quinonez et al. https://creativecommons.org/licenses/by/4.0/ This content is distributed under the terms of the Creative Commons Attribution 4.0 International license.

10.1128/mbio.03559-21.4 FIG S4 NADH/NAD ratio level of the Erdman wild-type (WT), *ΔpckA*, and COM strains in 0.2% (wt/vol) acetate (A) or 0.2% (vol/vol) glycerol (B) medium. (C) Shown are intrabacterial NADH levels in WT, Δ*pckA*, and COM cells after 2 days. NADH levels were measured with a fluorescent NAD/NADH detection kit. All values are the average of independent triplicates ± SEM. *, *P* < 0.05, and ***, *P* < 0.001, by Student’s unpaired *t* test. Download FIG S4, TIF file, 2.7 MB. Copyright © 2022 Quinonez et al. 2022 Quinonez et al. https://creativecommons.org/licenses/by/4.0/ This content is distributed under the terms of the Creative Commons Attribution 4.0 International license.

### Acetate-induced nonreplication in the Δ*pckA* mutant increases drug tolerance against INH and BDQ.

**FIG 2:**
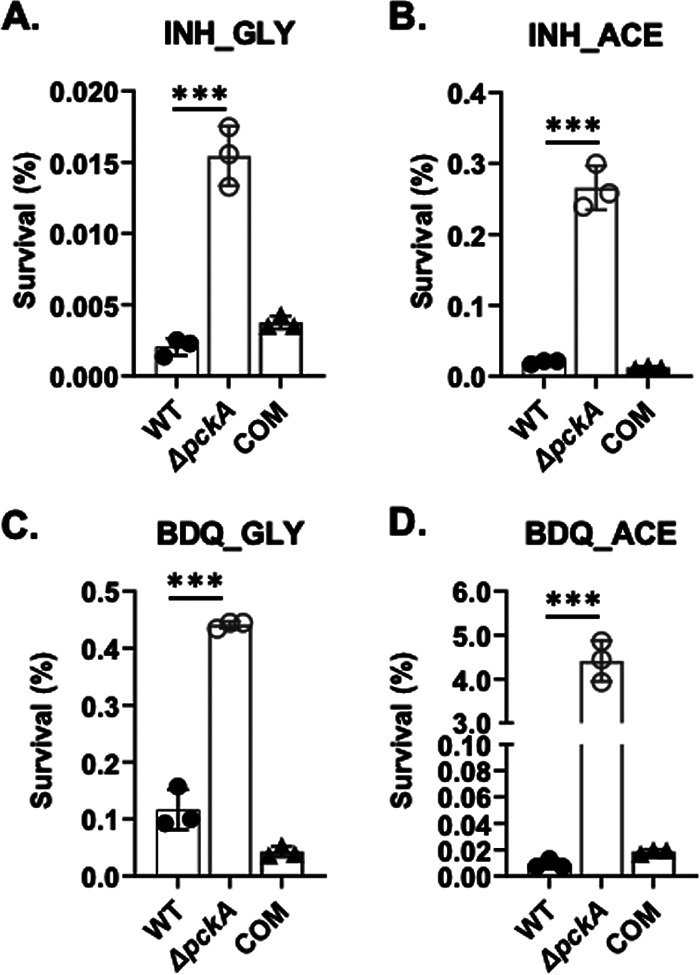
Percentage of survival rates of the WT, Δ*pckA*, and COM strains in glycerol or acetate medium in response to treatment with isoniazid (INH) or bedaquiline (BDQ). CFU viability of the three strains after treatment with 0.3 μg/mL of INH (A and B) and BDQ (C and D) was calculated after a 5-day or 7-day incubation, respectively. The percentage of survival was calculated by the CFU at the final time point relative to input CFU. Three strains were cultured in medium containing glycerol (GLY [A and C]) or acetate (ACE [B and D]). All values are the average of experimental triplicates ± SEM and representative of at least two independent experiments. ***, *P* < 0.001 by Student’s unpaired *t* test.

Given the carbon source-dependent metabolic remodeling and phenotypic changes of the Δ*pckA* mutant, we explored the drug tolerance of the Δ*pckA* mutant against the clinically relevant anti-TB drugs INH and BDQ. The WT, Δ*pckA*, or COM strain was treated with 10× the MIC of both INH and BDQ for 5 or 7 days, respectively, in glycerol or acetate media. The cells were serially diluted and plated on m7H10, and the colonies were counted after 3 weeks of incubation at 37°C. The Δ*pckA* mutant was able to maintain a greater level of viable colonies after treatment with first- and second-line TB drugs in both carbon sources, albeit the level was much greater in acetate medium (Fig. 2A to D). The percentage of survival rates of the Δ*pckA* mutant in acetate medium were significantly greater by ∼13-fold (0.26% versus 0.02%) under the INH treatment condition or ∼487-fold (4.4% versus 0.0009%) under the BDQ treatment condition compared to those of the WT (Fig. 2B and D). These findings demonstrated that the acetate-induced bacteriostatic phenotypes and accompanying metabolic remodeling of the Δ*pckA* mutant led to a greater level of drug tolerance against conventional TB drugs (Fig. 2B and D; see Fig. S5A and B in the supplemental material). These findings also confirmed that *M. tuberculosis* cells in a state of viable but reduced growth rate are normally less susceptible to anti-TB drugs. Intriguingly, WT cells, despite an intact PEPCK activity, became significantly more tolerant to DCS and INH when cultured in acetate medium compared to glycerol medium (Fig. 1A; Fig. S5A to C).

10.1128/mbio.03559-21.5 FIG S5 (A and B) Various concentrations of INH killing curves of the WT, Δ*pckA*, and COM strains in media containing 0.2% (vol/vol) glycerol (A) and 0.2% (wt/vol) acetate (B). Drug tolerance in an INH concentration-dependent manner (1× the MIC, 0.03 μg/mL; 10× the MIC, 0.3 μg/mL; 100× the MIC, 3 μg/mL). All values are the average of independent triplicates ± SEM. *, *P* < 0.05, and ***, *P* < 0.001, by ANOVA. Fold change was calculated based on colonies formed relative to those at day 0. (C) Killing curves of the WT after treatment with 100 μg/mL d-cycloserine (DCS) for 21 days. An exponentially growing population of WT cells was exposed to 0.2% (vol/vol) glycerol and 0.2% (wt/vol) acetate-containing media with 100 μg/mL DCS. Viable count (CFU/mL) results are shown. All values are the average of independent triplicates ± SEM. ***, *P* < 0.001 by Student’s unpaired *t* test. Download FIG S5, TIF file, 2.7 MB. Copyright © 2022 Quinonez et al. 2022 Quinonez et al. https://creativecommons.org/licenses/by/4.0/ This content is distributed under the terms of the Creative Commons Attribution 4.0 International license.

### Greater drug tolerance of the Δ*pckA* mutant correlates with overaccumulation of MCC intermediates.

**FIG 3:**
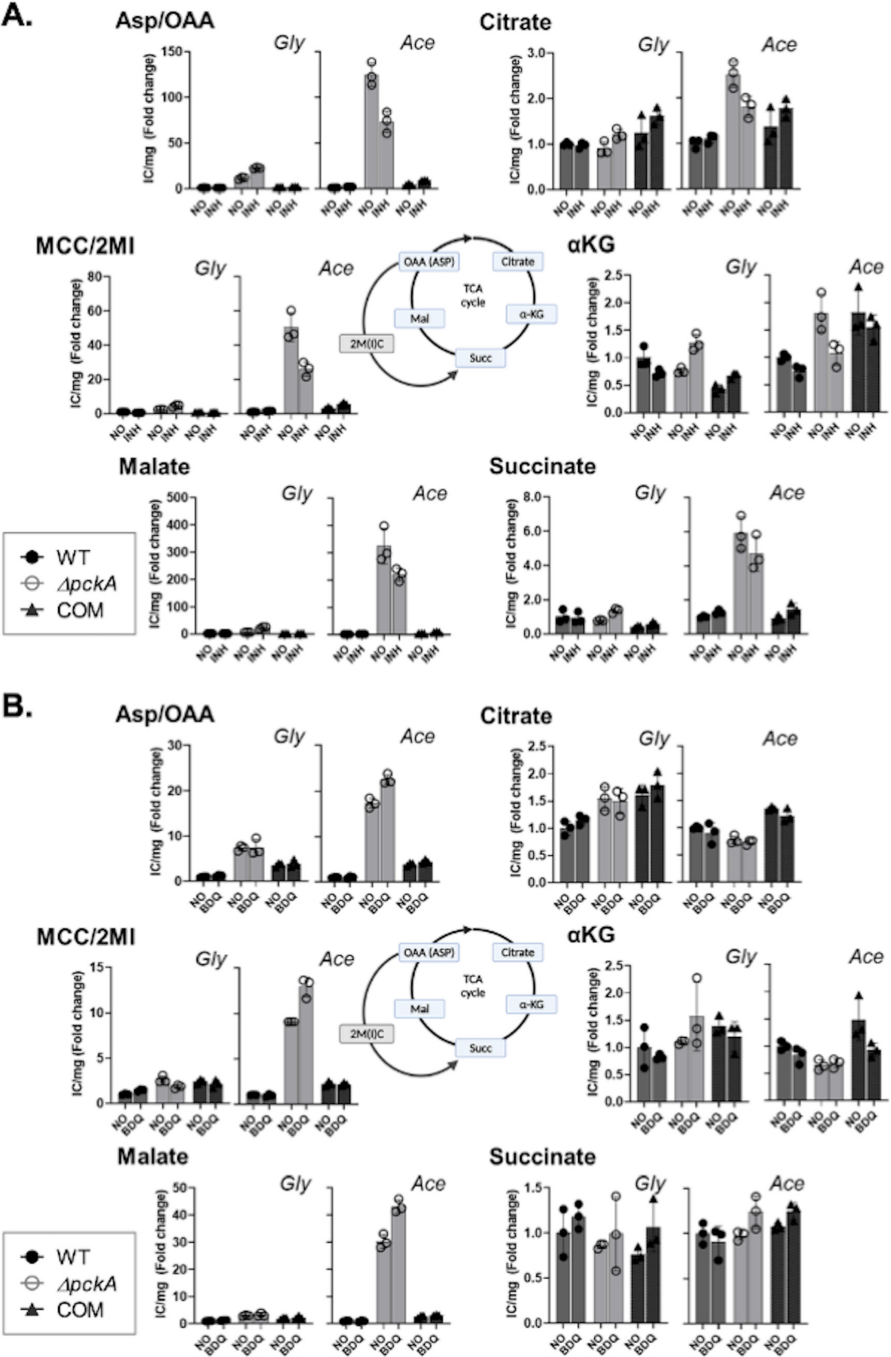
Targeted metabolomics analysis of the TCA cycle and MCC of the WT, Δ*pckA*, and COM strains under antibiotic treatment. Intrabacterial pool sizes of TCA cycle and MCC intermediates of the three strains cultured in glycerol (Gly) or acetate (Ace) medium were monitored after treatment with 0.3 μg/mL INH (A) or 0.3 μg/mL BDQ (B) for 24 h. Pool sizes are expressed in fold changes relative to the ion counts/mg protein of WT in the no-treatment control (*y* axis). α-KG, α-ketoglutarate; Succ, succinate; Mal, malate; OAA, oxaloacetate; ASP, aspartate; 2M(I)C, 2 methyl (iso)citrate. All values are the average of independent triplicates ± SEM.

We conducted a metabolomics analysis to investigate the role of fatty acid-induced metabolic changes of the Δ*pckA* mutant in drug tolerance. To this end, we generated *M. tuberculosis*-laden filters containing WT, Δ*pckA* mutant, or COM strain cultures in the mid-logarithmic phase and exposed them to 10× the MIC of INH or BDQ. After incubation for 24 h, the cells were harvested before the loss of viability, and the metabolome was sampled by mechanical lysis to allow targeted LC-MS metabolomics analysis focusing on intermediates in the TCA cycle and MCC (28). This analysis showed that intermediates accumulated in the reductive branch of the TCA cycle, such as malate and aspartate, and the MCC, such as 2-methylcitrate and 2-methylisocitrate, of the Δ*pckA* mutant in both glycerol and acetate media were maintained even after treatment with INH or BDQ (Fig. 3A and B). Similar patterns were also observed after treatment with DCS (see Fig. S6 in the supplemental material). Intriguingly, BDQ treatment further enhanced the abundance of the TCA cycle and MCC intermediates of the Δ*pckA* mutant, while only minor changes or slight downregulation was detected in response to treatment with INH or DCS, which was corroborated by the finding that the Δ*pckA* mutant was tolerant to BDQ much greater than to INH (Fig. 2B and D). Unlike the reductive TCA cycle branch or MCC intermediates, the oxidative TCA cycle branch intermediates of the Δ*pckA* mutant, such as citrate and α-KG, were unaltered or even further downregulated after antibiotic treatment relative to those of the Δ*pckA* mutant without treatment (Fig. 3A and B; Fig. S6). Bypassing the oxidative branch of the TCA cycle is known to be associated with enhanced glyoxylate shunt activity—an adaptive strategy of *M. tuberculosis* used to survive hypoxia or antibiotic effects (11, 15). These results showed that the metabolic profile of the acetate-induced Δ*pckA* nonreplicating phenotype (Fig. 1B; Fig. S2) was also maintained even when exposed to first- and second-line TB drugs (Fig. 3; Fig. S6), resulting in the high level of drug tolerance.

10.1128/mbio.03559-21.6 FIG S6 DCS induced remodeling of TCA cycle and MCC activity of the WT, Δ*pckA*, and COM strains. Shown are intrabacterial pool sizes of TCA and the MCC intermediates of the WT, Δ*pckA*, and COM strains in 0.2% (vol/vol) glycerol (Gly) or 0.2% (wt/vol) acetate (Ace) medium in the presence or absence of 100 μg/mL DCS for 24 h. Total bar heights indicate the intrabacterial concentration. α-KG, α-ketoglutarate; Succ, succinate; Mal, malate; OAA (ASP), oxaloacetate (aspartate); MCC/2MI, methylcitrate/2 methylisocitrate. All values are the average of independent triplicates ± SEM. Download FIG S6, TIF file, 2.7 MB. Copyright © 2022 Quinonez et al. 2022 Quinonez et al. https://creativecommons.org/licenses/by/4.0/ This content is distributed under the terms of the Creative Commons Attribution 4.0 International license.

### Impact on *M. tuberculosis* bioenergetics due to *pckA* deficiency after INH treatment.

**FIG 4:**
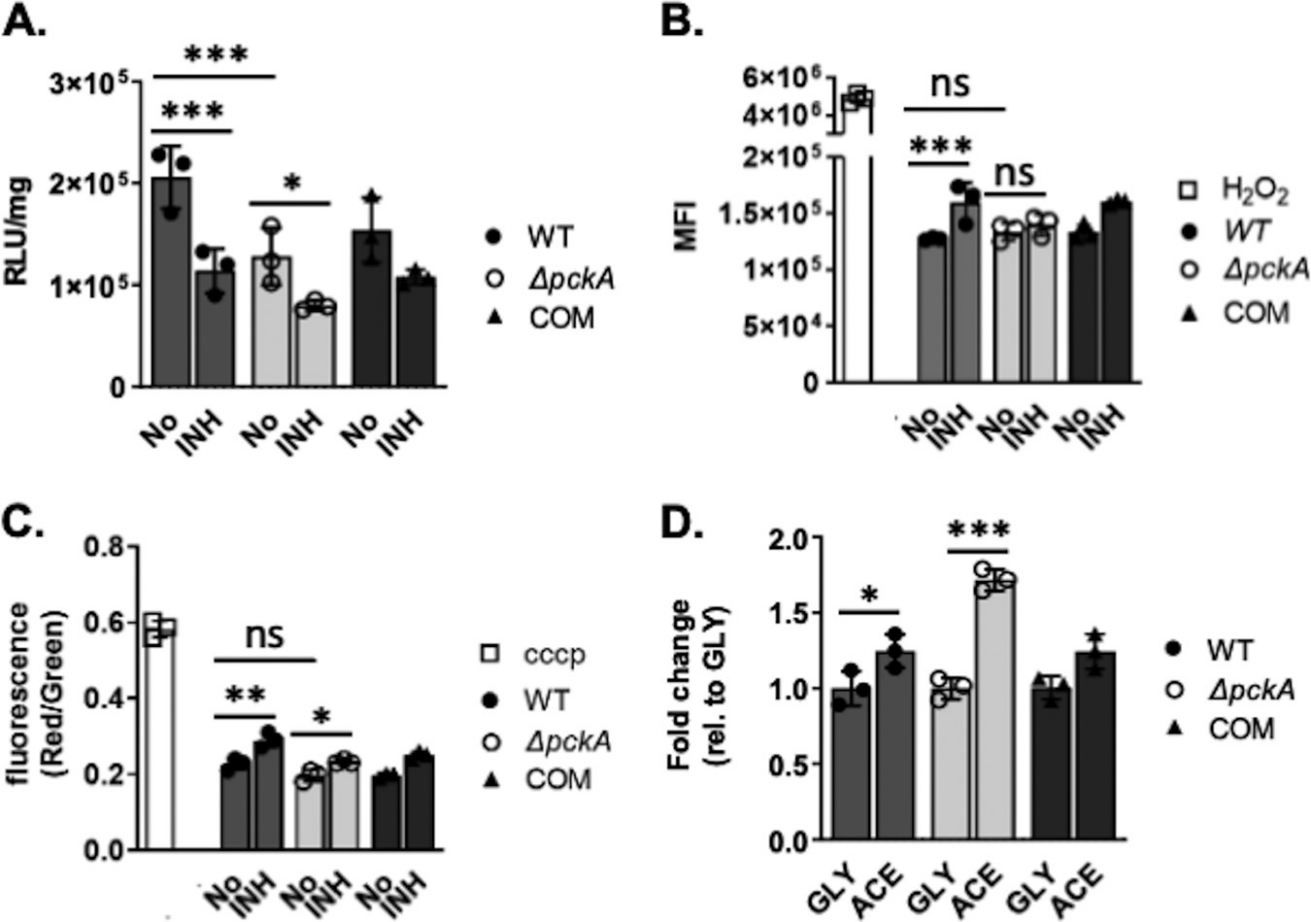
Impact on *M. tuberculosis* bioenergetics due to *pckA* deficiency after isoniazid (INH) treatment. WT, Δ*pckA*, and COM cells were treated with 0.3 μg/mL INH for 24 h. (A) Intrabacterial ATP pool sizes were measured by the BacTiter-Glo cell viability assay kit. (B) For reactive oxygen species (ROS), the strains were loaded with dihydroethidium, incubated with INH, and measured by flow cytometry. Treatment with 1 mM H_2_O_2_ was included as a positive control. (C) For membrane potential, the three strains were loaded with DiOC2(3), incubated with INH, and measured by flow cytometry. Treatment with 5 carbonyl cyanide *m*-chlorophenyl hydrazone (CCCP) was included as a positive control. Background fluorescence was subtracted from each value shown. (D) Acetate carbon-associated ROS levels of all strains relative to those in glycerol medium. All values are the average of independent triplicates ± SEM. ***, *P* < 0.001, **, *P* < 0.01, *, *P* < 0.05, and ns, not significant, by Student’s unpaired *t* test.

A previous study has shown that the accumulation of MCC intermediates causes dysregulated respiratory activity and subsequent destabilized membrane bioenergetics (29, 30). A separate study confirmed that the enhanced respiration rendered *M. tuberculosis* in a dormancy-like state to be metabolically active and hypersensitive to INH via induced oxidative damage (31). Thus, to determine if the remodeled membrane bioenergetics results in Δ*pckA* mutant tolerance against INH, we measured the sensitivity to reactive oxygen species (ROS), membrane potential, and ATP levels of all three strains in acetate m7H9 upon exposure to INH. ROS, membrane potential, and ATP levels were quantified by flow cytometry using specific fluorescence dyes before and after treatment with 10× the MIC of INH (29). BacLight DiOC_2_(3) membrane potential dye was used to detect membrane potential homeostasis. This dye accumulates inside bacteria and emits green fluorescence in direct proportion to size. Under active oxidative phosphorylation, this dye also emits red fluorescence. The red/green fluorescence ratio can be used to track changes in bacterial membrane potential. H_2_O_2_ and CCCP (carbonyl-cyanide 3-chlorophenylhydrazone) were used as controls for ROS and membrane potential, respectively. The results showed that the Δ*pckA* mutant produced less ATP, even under no antibiotic treatment (Fig. 4A), while maintaining ROS and membrane potential at levels similar to those of the WT or COM strain under acetate medium (Fig. 4B and C). Interestingly, the Δ*pckA* mutant already had significantly increased intrabacterial ROS content when cultured in acetate medium compared to when cultured in glycerol medium. The fold increase shown in Δ*pckA* strain ROS levels in acetate medium was significantly greater than that in glycerol medium demonstrated by the WT when comparing ROS levels in acetate to those in glycerol (Fig. 4D). Intriguingly, ATP levels of the Δ*pckA* strain were not as negatively impacted by INH treatment as those of the WT, and the ROS levels of the Δ*pckA* strain were also relatively unaffected by INH treatment (Fig. 4A and B). This may be largely due to the slowed TCA cycle activity and low levels of ETC activity. The membrane potential of the Δ*pckA* strain was also relatively unaltered by treatment with INH compared to that of WT (Fig. 4C), presumably due to the presence of an additional strategy to sustain the membrane potential, including the active secretion of succinate, as previously reported (11, 32). Notably, a greater amount of succinate was secreted by Δ*pckA* cells cultured in acetate m7H9 compared to that of either WT cells in acetate m7H9 or Δ*pckA* cells in glycerol m7H9 (see Fig. S7A in the supplemental material). Consistent with previous reports, the MCC intermediates, if accumulated, affect the respiratory activity and membrane bioenergetics, leading to the growth arrest and drug tolerance (29).

10.1128/mbio.03559-21.7 FIG S7 (A) Secretion of succinate following incubation of WT, Δ*pckA*, and COM cells in medium containing glycerol (GLY) or acetate (ACE). Total bar heights indicate the concentration of secreted succinate. All values are the average of independent triplicates ± SEM. *, *P* < 0.05, and ns, not significant, by Student’s unpaired *t* test. (B and C) The effect of PEPCK deficiency on PEP biosynthesis using acetate as a carbon source. (B) Intrabacterial pool sizes and ^13^C enrichment of PEP of WT, Δ*pckA*, and COM cells incubated in ^13^C fully labeled [U-^13^C]acetate-containing medium for 24 h. Total bar heights indicate the intrabacterial PEP abundance, whereas the white area of each bar denotes the extent of ^13^C labeling achieved following transfer to [U-^13^C]acetate. (C) Schematic depicting *M. tuberculosis* CCM, including glycolysis and the TCA cycle. Gluconeogenesis is denoted by green arrows, glycolysis is denoted by gray arrows, and the TCA cycle is denoted by black arrows. PEPCK is involved in the first step of gluconeogenesis converting OAA to PEP. PEP, phosphoenolpyruvate; α-KG, α-ketoglutarate; Succ, succinate; Mal, malate; OAA, oxaloacetate. PEPCK converts OAA to PEP. In the Δ*pckA* mutant, this reaction is not present, and thus, there should be no PEP when acetate is the sole carbon source. Surprisingly, when cultured in medium containing [U-^13^C]acetate, the Δ*pckA* mutant was able to biosynthesize unlabeled PEP (only labeled with ^12^C), implying the presence of endogenous carbon sources possibly used to biosynthesize PEP. Thus, the PEP level was almost as abundant as it was in the WT. The majority of the PEP in the WT or COM strain was labeled, indicating that both strains used acetate as a source of PEP. Download FIG S7, TIF file, 0.2 MB. Copyright © 2022 Quinonez et al. 2022 Quinonez et al. https://creativecommons.org/licenses/by/4.0/ This content is distributed under the terms of the Creative Commons Attribution 4.0 International license.

### The ATP level of the Δ*pckA* mutant is unaffected by BDQ treatment.

**FIG 5:**
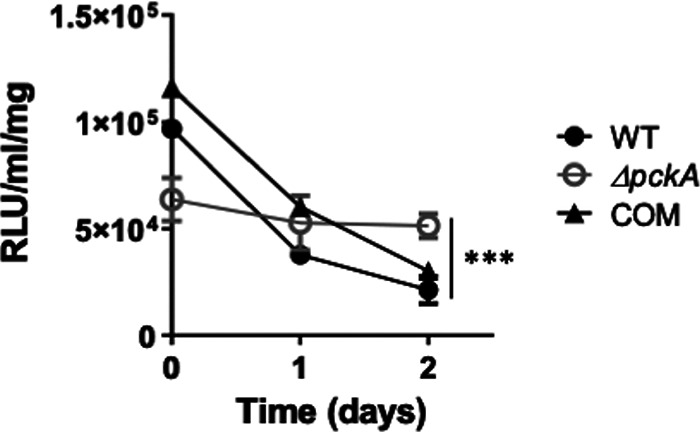
Kinetics of ATP levels of the WT, Δ*pckA*, and COM strains following treatment with bedaquiline (BDQ). ATP kinetics were monitored over 2 days in acetate medium after treatment with 0.3 μg/mL BDQ. Intrabacterial ATP content was measured by BacTiter-Glo cell viability assay kit. Data represent an average of biological triplicates ± SEM. ***, *P* < 0.001 by ANOVA.

BDQ inhibits the c and ɛ subunits of *M. tuberculosis* ATP synthase (33–36). Therefore, we were interested in understanding if ATP levels of the Δ*pckA* mutant were affected in acetate medium when exposed to this drug. In the absence of BDQ treatment in acetate m7H9, the ATP abundance of the Δ*pckA* mutant was maintained at levels significantly lower than those of the other two strains (Fig. 4A and Fig. 5), which correlates with the dormancy-like state, as previously identified (27, 37). Intriguingly, when exposed to BDQ, the ATP levels were not significantly altered at least for 2 days, while the ATP levels of WT and COM rapidly decreased (Fig. 5). This rapid decrease in ATP levels of the WT and COM strains, not of the Δ*pckA* mutant, suggested that the Δ*pckA* mutant largely relies on metabolic sources other than ETC activity for ATP biosynthesis. Thus, the Δ*pckA* mutant in the acetate medium could maintain its ATP levels despite the treatment with high doses of BDQ. Collectively, the findings suggested that the accumulation of MCC intermediates downregulates the ETC activity, while other metabolic activities may be involved in maintaining the ATP pool, such as the activation of trehalose metabolism that feeds toward glycolysis (12, 13, 38) or possibly slowed kinetics of the ATP consumption rate. To interrogate the role of preexisting carbon sources to feed glycolysis, we proceeded to calculate ^13^C enrichment rates of Δ*pckA* mutant PEP by transferring cells from medium containing unlabeled acetate to medium containing [U-^13^C]acetate, as was done to study ^13^C labeling patterns of TCA cycle intermediates (Fig. S3). We observed that the Δ*pckA* mutant biosynthesized PEP, the end product of *M. tuberculosis* glycolysis, at levels similar to those of the WT or COM strain, and a significant portion of the PEP biosynthesis occurred within the unlabeled fraction (Fig. S7B and C). Biosynthesis of PEP in the WT or COM strain, however, occurred within the ^13^C-labeled fraction, implying that the WT or COM strain efficiently consumed [U-^13^C]acetate, but the Δ*pckA* mutant used unknown internal sources (Fig. S7B). These findings collectively suggested the existence of endogenous carbon sources and the role of substrate-level phosphorylation of *M. tuberculosis* glycolysis activity in maintaining ATP levels of the Δ*pckA* mutant upon treatment with BDQ.

### Overaccumulation of MCC intermediates is advantageous to achieve drug tolerance in *M. tuberculosis*.

**FIG 6:**
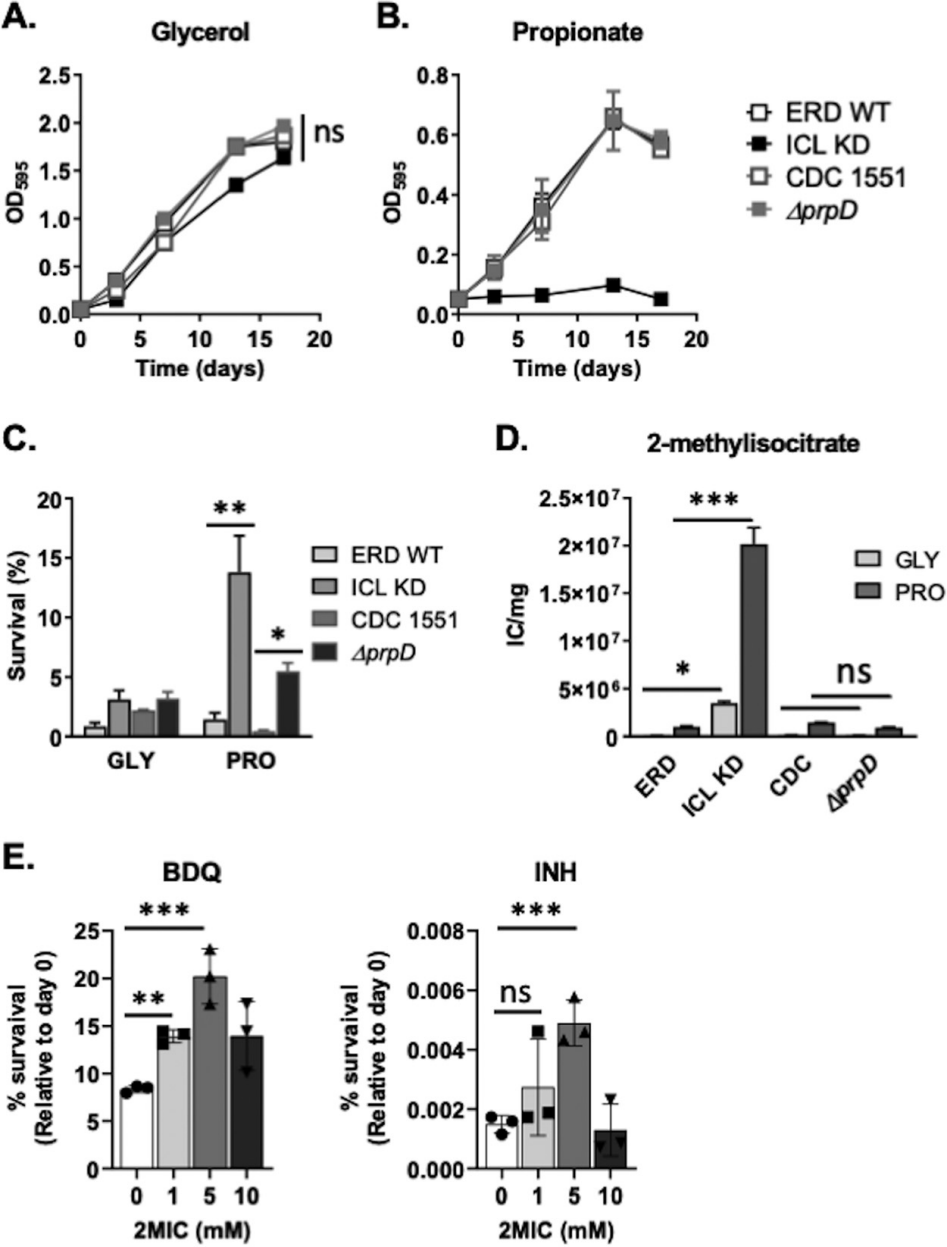
The effects of overaccumulation of MCC intermediates on drug tolerance. Growth kinetics of the ICL KD strain (and its parental strain, Erdman) and Δ*prpD* mutant (and its parental strain, CDC1551) in glycerol medium (A) or propionate medium (B) were monitored by OD_595_. All values are the average of independent triplicates ± SEM. ns, not significant by ANOVA. (C) Percentage of survival rates of the *M. tuberculosis* strains in glycerol (GLY) or propionate (PRO) medium were measured after treatment with 0.3 μg/mL INH for 5 days. The percentage of survival was calculated by the CFU at the final time point relative to input CFU. (D) Intrabacterial 2-methylisocitrate pool sizes of each strain cultured in glycerol or propionate medium were monitored by LC-MS. (E) The effects of authentic 2MIC (2-methylisocitrate) on *M. tuberculosis* drug tolerance were assessed by monitoring the CFU viability of the Erdman WT strain in glycerol medium using 0.3 μg/mL BDQ (left panel) or 0.3 μg/mL INH (right panel). Various doses (0, 1, 5, and 10 mM) of 2MIC were used for cotreatment. All values are the average of independent triplicates ± SEM. *, *P* < 0.05, **, *P* < 0.01, ***, *P* < 0.001, and ns, not significant, by Student’s unpaired *t* test.

To determine if the MCC intermediate accumulation may indeed be associated with drug tolerance, we used two mutants lacking enzymes in the MCC: an isocitrate lyase knockdown (ICL KD) strain and a 2-methylcitrate dehydratase knockout (Δ*prpD*) strain. ICL catalyzes the last step (2-methylisocitrate to succinate and pyruvate) of the MCC (Fig. 1C, right panel) (29, 39). The ICL KD strain is a conditional knockdown mutant generated in the Schnappinger laboratory by adapting the MultiSite Gateway recombinational cloning method to assemble a regulated expression plasmid adapted to expression in *M. tuberculosis* (40). The ICL KD strain is a TetON mutant, and its expression is induced by treatment with anhydrotetracycline (ATc). The 2-methylcitrate dehydratase (PrpD) catalytically converts 2-methylcitrate to 2-methyl *cis*-aconitate. Previously, ICL-deficient *M. tuberculosis* had been shown to have a growth defect in fatty acid medium, and thus, ICL was considered to be one of the best drug targets (24). As propionate is an initial substrate of the *M. tuberculosis* MCC, we assessed the growth kinetics of the ICL KD and Δ*prpD* strains together with their parental strains (Erdman and CDC1551, respectively) in glycerol and propionate media as the sole carbon sources. The ICL KD strain’s growth kinetics were almost identical to those of the WT when cultured in glycerol m7H9 due to the functional dispensability of ICL activity in consuming glycerol as a carbon source (Fig. 6A). In propionate m7H9, ICL KD strain growth was completely impaired in the absence of ATc but fully restored at a level similar to that of the WT by treatment with ATc (Fig. 6B; see Fig. S8A in the supplemental material). However, rather intriguingly, the Δ*prpD* mutant grew normally in both glycerol m7H9 and propionate m7H9 (Fig. 6A and B), which was somewhat different from the results reported by Muñoz-Elias et al., although the strain used in their study was Δ*prpDC* (41). These findings suggested that the buildup of either 2-methyl *cis*-aconitate or 2-methylisocitrate was toxic to *M. tuberculosis* growth. We then exposed these four strains (WT and mutants) to INH in either glycerol m7H9 or propionate m7H9 and monitored the viable colonies after a 5-day incubation. Surprisingly, the ICL KD strain had a better survival rate than the other strains, which were restored by treatment with ATc (Fig. 6C; Fig. S8B). Similar to the Δ*pckA* mutant, only the ICL KD strain showed a significant accumulation of MCC intermediates in both glycerol and propionate media, albeit much greater in propionate medium (Fig. 6D; Fig. S8C). Interestingly, unlike the metabolomics profile of the Δ*pckA* mutant, the ICL KD mutant did not accumulate TCA cycle intermediates, except for succinate (Fig. S8C); thus, the tolerance is likely to be mediated by the accumulation of MCC intermediates. To confirm the role of MCC intermediates in *M. tuberculosis* drug tolerance, we chemically synthesized 2-methylisocitrate as conducted in our previous report (30), treated the Erdman WT strain with various doses of authentic 2-methylisocitrate (1 to 10 mM), and monitored the drug sensitivity against INH or BDQ. The percentage of survival rates indicated that 5 mM 2-methylisocitrate was sufficient to partly protect *M. tuberculosis* from the antibiotic effects of both INH and BDQ by ∼2- to 3-fold compared to the rate without treatment with 2-methylisocitrate (Fig. 6E). Concentrations of 2-methylisocitrate at greater than 10 mM might be toxic to *M. tuberculosis* viability (Fig. 6E). These findings are also corroborated by the percentage of survival rate of the ICL KD strain without ATc against INH (Fig. 6C and D; Fig. S8B).

10.1128/mbio.03559-21.8 FIG S8 Phenotypic characterization of ICL knockdown (KD). (A) Growth kinetics of the ERD WT and ICL KD strains with or without 1 μg/mL of anhydrotetracycline (ATc) in 0.1% (wt/vol) propionate-containing medium. (B) CFU viability of the WT and ICL KD strains in the presence or absence of 1 μg/mL of ATc in 0.1% (wt/vol) propionate medium after treatment with 10× the MIC of isoniazid (INH) for 5 days. The percentage of survival was calculated by the number of colonies at the fifth day under the INH-treated condition and the initial number of colonies before INH treatment. (C) Metabolomics analysis of the WT, ICL KD, CDC1551, and Δ*prpD* strains cultured in medium containing either glycerol or propionate as the sole carbon source. The analysis was focused on intermediates in the TCA cycle and MCC. Total bar heights indicate the intrabacterial concentration. α-KG, α-ketoglutarate; Succ, succinate; Mal, malate; OAA (ASP), oxaloacetate (aspartate); MCC, 2 methylisocitrate. All values are the average of independent triplicates ± SEM. ***, *P* < 0.001 by Student’s unpaired *t* test. Download FIG S8, TIF file, 2.4 MB. Copyright © 2022 Quinonez et al. 2022 Quinonez et al. https://creativecommons.org/licenses/by/4.0/ This content is distributed under the terms of the Creative Commons Attribution 4.0 International license.

## DISCUSSION

Carbon metabolism is a significant determinant of *M. tuberculosis*’s ability to replicate and persist within the host (21, 23). Defining the metabolic pathways of *M. tuberculosis* used to adapt to the host’s carbon environment is essential to understand its pathogenicity and to act as a guide for the development of new therapeutic options. Much attention has been focused on the glyoxylate shunt since it has been shown that *M. tuberculosis* relies on this pathway as a fatty acid catabolism route for *in vivo* growth (28, 42, 43) and virulence (39), where isocitrate lyase 1 is a key to initiate the activity (41). The same enzyme also plays a role in the last step of the MCC, a pathway thought to break down toxic compounds derived from propionate metabolism (29). Here, we investigated the role of fatty acid metabolism in drug tolerance as it is the main carbon source available within the host during the acute and chronic phases of *M. tuberculosis* infection (11, 23, 44). We identified that metabolic networks used to consume fatty acids include the MCC by conducting metabolomics profiling of Δ*pckA* and ICL KD strains. At a high level of 2-methylisocitrate accumulation, this pathway was reported to acidify the intracellular pH that subsequently activates the glutamate-GABA conversion activity as a mechanism to neutralize the proton buildup caused by hyperactive propionate metabolism (29, 30). Intriguingly, the fatty acid-triggered overaccumulation of MCC intermediates may be advantageous to achieve drug tolerance when treated with high doses of antibiotics. Notably, treatment with authentic 2-methylisocitrate improved the survival rates of *M. tuberculosis* under antibiotic treatment even in glycerol medium (Fig. 6E). Furthermore, when cultured in acetate medium, the WT showed higher drug tolerance than in glycerol medium (Fig. 1A; Fig. S5), suggesting that *M. tuberculosis* metabolic networks required to consume acetate may be implicated in drug tolerance.

The accumulation of MCC and TCA cycle intermediates in the Δ*pckA* mutant occurred due to gluconeogenic carbon flux obstruction, which was accompanied by the slowdown of TCA cycle activity (23). *M. tuberculosis* treated with bactericidal TB antibiotics (including INH) previously reported by Nandakumar et al. elicited similar accumulations of reductive TCA cycle branch intermediates, such as succinate, fumarate, and malate, as well as a decrease in an oxidative TCA cycle intermediate, α-KG, in H37Rv (15). This study further showed that the remodeling in TCA cycle intermediates was largely due to induced glyoxylate shunt activity. Here, qRT-PCR, ^13^C isotopologue pattern analysis, and NADH/NAD quantitation confirmed that an accumulation of the TCA cycle intermediates of the Δ*pckA* strain accompanied by improved drug tolerance was attributed to systemic slowdown of TCA cycle activity and functional depletion of ETC activity (Fig. 1C; Fig. S3 and S4).

Dysregulated ETC activity is often associated with the accumulation of ROS, but Rowe et al. demonstrated that ROS also triggered the downregulation of TCA cycle activity and led to a drug-tolerant state (45). We observed that the Δ*pckA* mutant in acetate medium had increased ROS levels compared to that in glycerol medium, which was less affected even after treatment with TB antibiotics (Fig. 4B and D). This suggests that in *pckA* deficiency, metabolic networks may be remodeled to neutralize the toxicity of increased ROS levels. This may include enhanced succinate secretion or unknown carbon mobilization through glycolysis (Fig. S7). It remains to be demonstrated if these alterations are responsible for the improved drug tolerance of the Δ*pckA* mutant in acetate medium.

The impact of the downregulated ETC activity on drug tolerance of *M. tuberculosis* against INH was investigated as previously described (31) (Fig. 4; Fig. S4). Thus, the acetate consumption mediated TCA cycle slowdown, altered ETC activity, induced ROS levels, and depleted ATP levels of the Δ*pckA* mutant, which collectively can be interpreted as a bioenergetic sign of dormancy-like stress, as also seen in hypoxic *M. tuberculosis* (11). This was a sharp contrast to the WT and COM strains as they were actively replicating in this carbon source.

Slowing down the TCA cycle activity together with accumulation of MCC intermediates caused a decrease in available reducing equivalents (NADH and FADH_2_) required to initiate the ETC activity, leading to reduced NAD recycling, respiration, and intracellular ATP. Even though the Δ*pckA* mutant had a lower TCA cycle activity (Fig. 1C; Fig. S3), intrabacterial ATP levels were maintained after 24 h of BDQ exposure (Fig. 5). The catalytic reaction of PEPCK requires ATP for the conversion of OAA to PEP in gluconeogenesis (23, 46). The lack of this pathway in the Δ*pckA* mutant presumably is an advantage as less ATP is consumed. Moreover, to overcome the absence of the gluconeogenesis pathway in acetate medium, the Δ*pckA* mutant may use preexisting endogenous carbon sources to support glycolysis (Fig. S7B and C). As glycolysis generates 15 times less ATP than oxidative phosphorylation, activation of glycolysis leads to a low ATP level, presumably inducing the dormancy-like state seen in the Δ*pckA* mutant. Thus, this remodeling of metabolism coupled with the dormant metabolic state that consumes less ATP can be a mechanistic basis that gives the Δ*pckA* strain the advantage to survive BDQ treatment (38). In the WT, the *pckA* mRNA levels are upregulated when gluconeogenesis is required (a pathway that consumes ATP). The WT cultured in acetate medium may facilitate the depletion of ATP levels in the presence of BDQ treatment compared to that of glycolytic carbon-containing medium, as previously confirmed (38, 47). Therefore, Δ*pckA* mutation may be advantageous to maintain ATP levels sufficient for higher bacterial viability than the WT. Thus, a similar adaptation was also seen for the Δ*pckA* mutant in acid growth arrest, where the strain also became tolerant to multiple antibiotics, including INH and rifampin (25). Our recent report separately raised another mechanism by revealing the impact of PEP depletion due to PEPCK deficiency on *M. tuberculosis* growth and drug tolerance because PEP plays a central role as a substrate to fuel multiple pathways required for active replication (26).

To provide direct evidence that accumulated MCC intermediates are involved in drug tolerance, we used Δ*prpD* and ICL KD strains, which are *M. tuberculosis* mutants that lack the first and last enzymes of the MCC, respectively. The ICL KD strain does not grow on fatty acids (29, 41); thus, to induce the dormancy-like state and examine the potential role of MCC overaccumulation, we provided propionate to both the ICL KD and Δ*prpD* strains rather than acetate to directly study MCC activity. The ICL KD strain accumulated MCC intermediates when consuming propionate as the sole carbon source. Unlike the metabolic state seen in the Δ*pckA* mutant, there was no accumulation of the TCA cycle metabolites (Fig. 6D; Fig. S8C), but the ICL KD strain showed greater levels of tolerance against INH in propionate medium compared to those of its parental strain, Erdman (Fig. 6C). Intriguingly, the Δ*prpD M. tuberculosis* mutant lacking an upstream enzyme in the MCC was also somewhat more tolerant to INH than its parental strain (CDC1551), albeit being less tolerant than the ICL KD strain, but the metabolomics profile showed no accumulation of MCC intermediates (Fig. 6D). Instead, we observed a significant accumulation of malate and aspartate (Fig. S8C). Aspartate plays a role in the biosynthesis of cofactors and peptidoglycan, the activity of which is essential for the thickening of *M. tuberculosis* cell wall (a known mechanism in drug tolerance). Moreover, a recent study demonstrated that the inhibition of the aspartate pathway leads to the clearance of chronic infection (48). Thus, the Δ*prpD* mutant’s tolerance to INH could be due to metabolic consequences arising from aspartate accumulation. It is worth noting that although the lack of PrpD enzyme should attenuate the strain’s growth in propionate medium, alternative routes for propionate oxidation, including the methylmalonyl pathway, have been previously suggested (41).

ICL has been identified as a promising drug target by several studies as the ICL knockout strain failed to survive under hypoxia (11), to maintain persistence and virulence in mice (44), and to be drug sensitive to a range of anti-TB drugs when tested in carbon-rich medium (15). Nonetheless, it was also known that *M. tuberculosis* in a nonreplicating state became drug tolerant (18, 49, 50), which was the case when the ICL KD and Δ*pckA* strains were exposed to model fatty acids. Under these experimental conditions, we observed that MCC intermediates played a vital role in the acquisition of high levels of drug tolerance; *M. tuberculosis* is exposed to a range of conditions inside the macrophages that are not tested here. Thus, the complex environment inside the macrophage yields an ICL-deficient strain unable to persist and establish infection. Thus, we maintain that ICL is a promising drug target, although we also acknowledge that an overaccumulation of MCC intermediates under specific experimental conditions may inversely invoke drug tolerance.

In summary, this work sheds light on carbon-induced drug tolerance’s involvement in the remodeling of central carbon metabolism. A possible mechanism is the overaccumulation of the MCC intermediates, leading to a series of metabolic changes and membrane bioenergetics aiding drug tolerance to both first- and second-line TB drugs. Understanding the versatility of the metabolic remodeling that *M. tuberculosis* can undergo with different carbon sources is important for the identification of drug targets and the development of new antibiotics.

## MATERIALS AND METHODS

### Bacterial strains and culture conditions.

The Mycobacterium tuberculosis Erdman wild-type strain (WT), Erdman *ΔpckA* mutant, Erdman *pckA* complemented (COM) strain, Erdman *icl* knockdown (ICL KD) strain, and CDC1551 and CDC1551 *ΔprpD* strains were precultured in Middlebrook 7H9 (m7H9) broth (Difco, Detroit, MI) supplemented with 0.5% (wt/vol) fraction V bovine serum albumin (BSA), 0.085% (wt/vol) NaCl, and 0.04% (vol/vol) tyloxapol with 0.2% (vol/vol) glycerol and 0.2% (wt/vol) dextrose. For experiments, the strains were resuspended in fresh m7H9 containing BSA, NaCl, tyloxapol, and 0.2% (vol/vol) glycerol, 0.2% (wt/vol) acetate, or 0.05 to 0.1% (wt/vol) propionate. The following antibiotics were added when necessary: isoniazid and bedaquiline at a final concentration of 0.3 μg/mL (10×) or 3 μg/mL (100×) and d-cycloserine at a final concentration of 100 μg/mL. For metabolomic profiling, filters were generated as previously described (8, 11). The *M. tuberculosis* Erdman and CDC1551 strains were cultured under containment in a biosafety level 3 facility. The *ΔprpD* mutant was purchased from BEI Resources.

### Bacterial growth curves and CFU assay.

Bacterial growth was monitored by optical density at 595 nm (OD_595_) by using a Genesys 20 spectrophotometer (Thermo Scientific). For CFU assays, cells in the mid-logarithmic growth phase of *M. tuberculosis* Erdman or CDC1551 were diluted to an OD_595_ of 0.05 in m7H9 broth containing BSA, NaCl, and tyloxapol and supplemented with 0.2% (vol/vol) glycerol, 0.2% (wt/vol) acetate, or 0.05 to 0.1% (wt/vol) propionate and antibiotics (isoniazid, bedaquiline, or d-cycloserine) when applicable, in a 24- or 96-well plate. After 5 or 7 days of antibiotic treatment (isoniazid and bedaquiline, respectively, or 7 and 14 days for d-cycloserine), the cells were then serially diluted and plated on m7H10 agar with 0.2% (vol/vol) glycerol, 0.2% (wt/vol) dextrose, 0.5 g/L BSA, and 0.085% (wt/vol) NaCl for 3 weeks at 37°C until the colonies were formed and counted. The ICL KD strain was plated on m7H9 containing 0.5% (vol/vol) glycerol, 0.2% (wt/vol) dextrose, 0.5 g/L BSA, 0.085% (wt/vol) NaCl, and 20 g/L agar due to its inability to grow on m7H10 medium containing malachite green.

### Metabolite extraction for LC-MS analysis.

The filters containing the Erdman or CDC1551 strains were incubated at 37°C. After reaching the mid-logarithmic phase of growth, the filters were transferred to chemically identical m7H10 agar containing fresh carbon source(s) and antibiotics when applicable and incubated for 24 h at 37°C. The metabolites were harvested by transferring the filters into precooled −40°C LC-MS-grade acetonitrile-methanol-water (40:40:20) solution and mechanically lysed with 0.1-mm Zirconia beads in a Precellys tissue homogenizer (Bertin Technologies, France) at 6,800 rpm for 6 min in dry ice. The lysate was centrifuged and filtered using 0.22-μm Spin-X columns (Sigma-Aldrich). The protein concentration of metabolite extracts was measured with a bicinchoninic acid (BCA) protein assay kit (Thermo Scientific, Waltham, MA, USA) to normalize samples to cell biomass.

### LC-MS for metabolomics profiling.

LC-MS differentiation and detection of Erdman, CDC1551, and mutant strains were performed with an Agilent Accurate mass 6230 time of flight (TOF) device coupled with an Agilent 1290 liquid chromatography system using solvents and configuration as previously described (11, 13). An isocratic pump was used for continuous infusion of a reference mass solution to allow mass axis calibration. Detected ions were classified as metabolites based on unique accurate mass-retention time identifiers for masses showing the expected distribution of accompanying isotopologues. Metabolites were analyzed using Agilent Qualitative Analysis B.07.00 and Profinder B.06.00 software (Agilent Technologies, Santa Clara, CA, USA) with a mass tolerance of <0.005 Da. Standards of authentic chemicals of known amounts were mixed with bacterial lysates and analyzed to generate the standard curves used to quantify metabolite levels.

### Isotopologue data analysis using isotope-labeled carbon source.

The extent of isotopic labeling for metabolites was determined by dividing the sum of the peak height ion intensities of all labeled isotopologue species by the ion intensity of both labeled and unlabeled species, expressed as a percentage. Label-specific ion counts were corrected for naturally occurring ^13^C species (i.e., [M + 1] and [M + 2]). The relative abundance of each isotopic form was represented by the sum of the peak 8 ion intensity of all labeled species.

### RNA extraction for qRT-PCR.

The Δ*pckA* mutant was grown on culture filter membranes as done in metabolite extraction and exposed to glycerol or acetate as the sole carbon source for 24 h. The total RNA was extracted using TRIzol solution (Sigma-Aldrich) and mechanically lysed with 0.1-mm Zirconia beads in a Precellys tissue homogenizer. Lysates were clarified by centrifugation, and the TRIzol supernatant was removed and used for RNA extraction. RNA was isolated using a Qiagen RNA extraction kit. Isolated RNA was treated with DNase I (Sigma-Aldrich) to remove DNA contamination (Sigma-Aldrich). RNA concentrations were determined using a Nanodrop spectrophotometer, and qRT-PCRs were conducted using an iQ SYBR green Supermix (Bio-Rad) and C1000 thermal cycler instrument (Bio-Rad). The primers used for amplification are listed in Table S1 in the supplemental material. Fold changes were calculated by values that were normalized to *sigA* transcript levels.

10.1128/mbio.03559-21.9 TABLE S1 Primer sets for qRT-PCR used in this study. Download Table S1, TIF file, 1.5 MB. Copyright © 2022 Quinonez et al. 2022 Quinonez et al. https://creativecommons.org/licenses/by/4.0/ This content is distributed under the terms of the Creative Commons Attribution 4.0 International license.

### Measurement of intrabacterial ATP levels.

Intrabacterial ATP concentrations were measured by BacTiter-Glo microbial cell viability assay (Promega) according to the manufacturer’s instructions. Cells were grown until an OD_595_ of 1.0 and diluted to 0.6 in fresh m7H9 medium containing the appropriate carbon source (glycerol or acetate) and antibiotic (bedaquiline or isoniazid). A 1-mL sample was taken at each time point, harvested, resuspended in 1 mL phosphate-buffered saline (PBS), and heat lysed at 100°C for 1 h. The lysate was centrifuged and filtered using a 0.22-μm Spin-X column, and the ATP was analyzed according to the manufacturer’s instructions (Thermo Fisher Scientific).

### ROS and membrane potential quantification.

Cells were grown until an OD_595_ of 1.0 and diluted to 0.6 in fresh m7H9 containing the appropriate carbon source (glycerol or acetate) and antibiotic (bedaquiline or isoniazid). A 1-mL sample was harvested and resuspended in 1 mL PBS with 0.04% (vol/vol) tyloxapol. For ROS quantification, the Total reactive oxygen species (ROS) assay kit, 520 nm (Thermo Fisher Scientific), was used. The cells were fixed with 2.5% (vol/vol) glutaraldehyde and stained with 10 μM (final) dihydroethidium for 1 h. The flow cytometry was set up according to the manufacturer’s instructions (Thermo Fisher Scientific). Positive (treated with 1 mM H_2_O_2_) and negative (untreated) controls were included. For membrane potential quantification, the instructions of the BacLight bacterial membrane potential kit (B34950; Molecular Probes) were followed. The cells were stained with 30 μM (final concentration) DiOC2(3) for 5 min, washed with PBS, and fixed with 2.5% (vol/vol) glutaraldehyde. A 5 μM concentration of carbonyl cyanide *m*-chlorophenyl hydrazine (CCCP) or unstained cells was included as a positive control and negative control, respectively.

### Succinate secretion measurement.

Our filter culture system was modified by replacing the underlying m7H10 agar with a plastic inset containing chemically equivalent m7H9 in direct contact with the underside of *M. tuberculosis*-laden filters, as previously conducted (11). m7H10 and m7H9 contained glycerol or acetate as a single carbon source. A blank filter was used as a negative control. After a 1-day incubation, cell-free m7H9 was collected, and the metabolome was extracted by adding an LC-MS-grade acetonitrile-methanol-H_2_O (40:40:20) solution, which was precooled to −40°C, and quantified by LC-MS. Total *M. tuberculosis* biomass was determined to normalize the succinate ion counts to biomass (BCA protein assay kit; Thermo Scientific).

### Statistical analysis.

Statistical analyses were performed by analysis of variance (ANOVA) and unpaired Student's *t* test. *P* values of <0.05 were considered statistically significant.
