# Supplementary figures and images for: The Role of Fatty Acid Metabolism in Drug Tolerance of Mycobacterium tuberculosis
Source: mBio. 2022 Jan 11;13(1):e03559-21. doi: 10.1128/mbio.03559-21 (PMC8749430; doi:10.1128/mbio.03559-21)

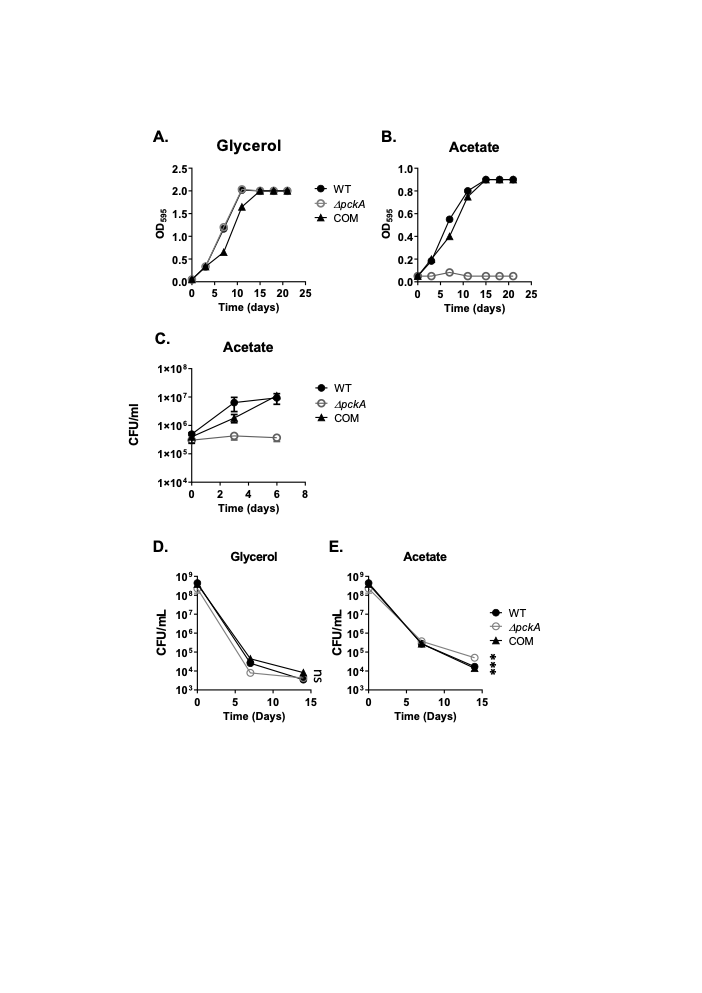

Supplement: FIG S1 [file mbio.03559-21-sf001.tif]

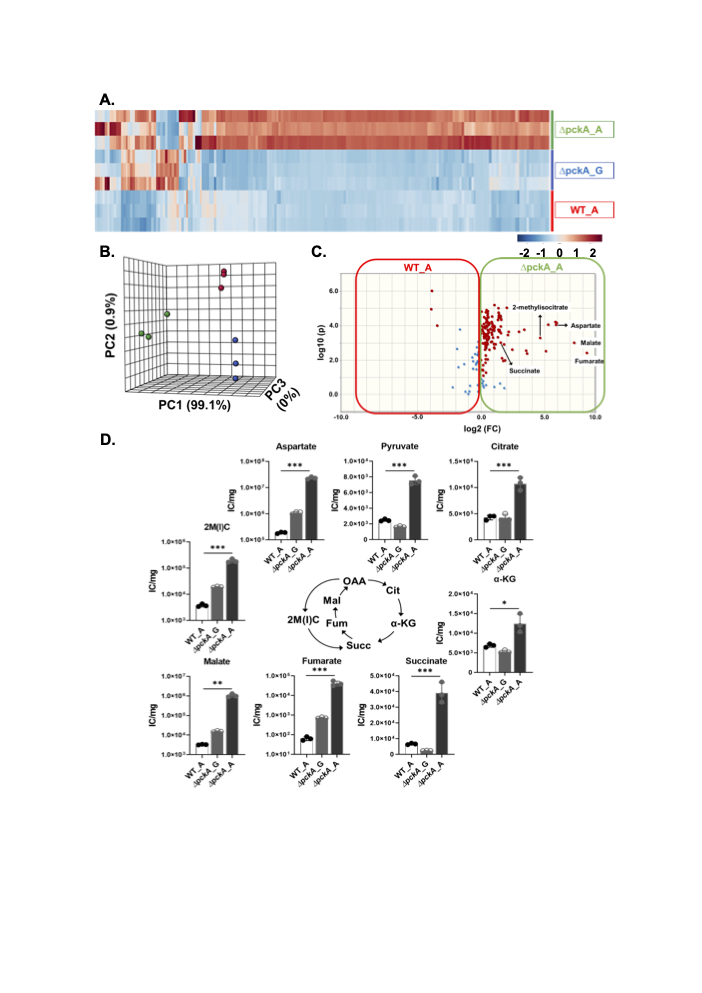

Supplement: FIG S2 [file mbio.03559-21-sf002.tif]

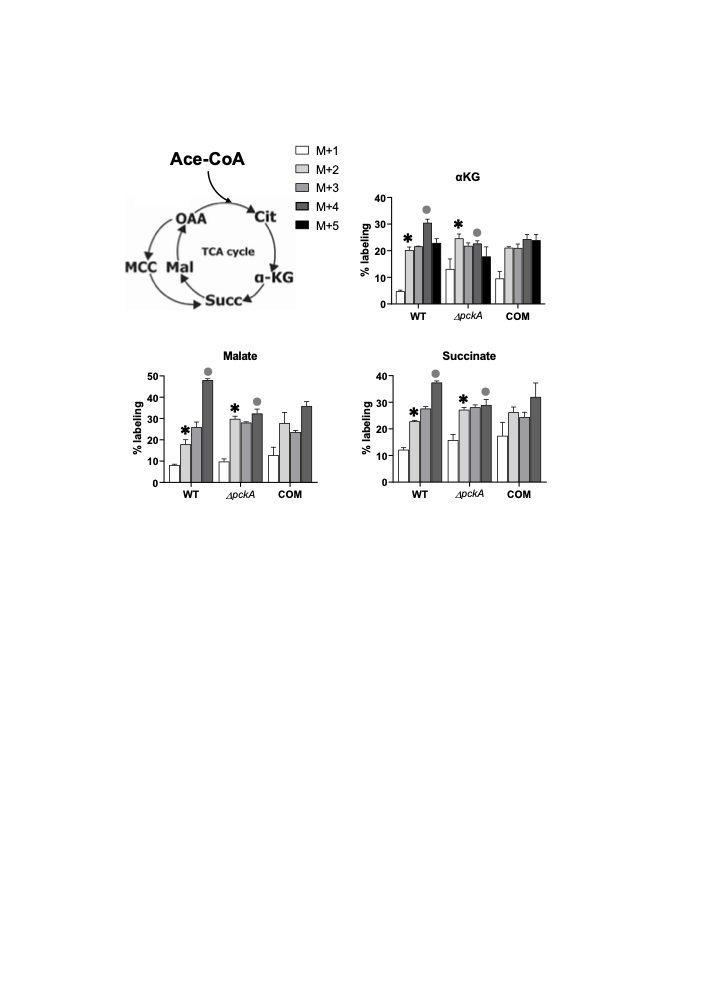

Supplement: FIG S3 [file mbio.03559-21-sf003.tif]

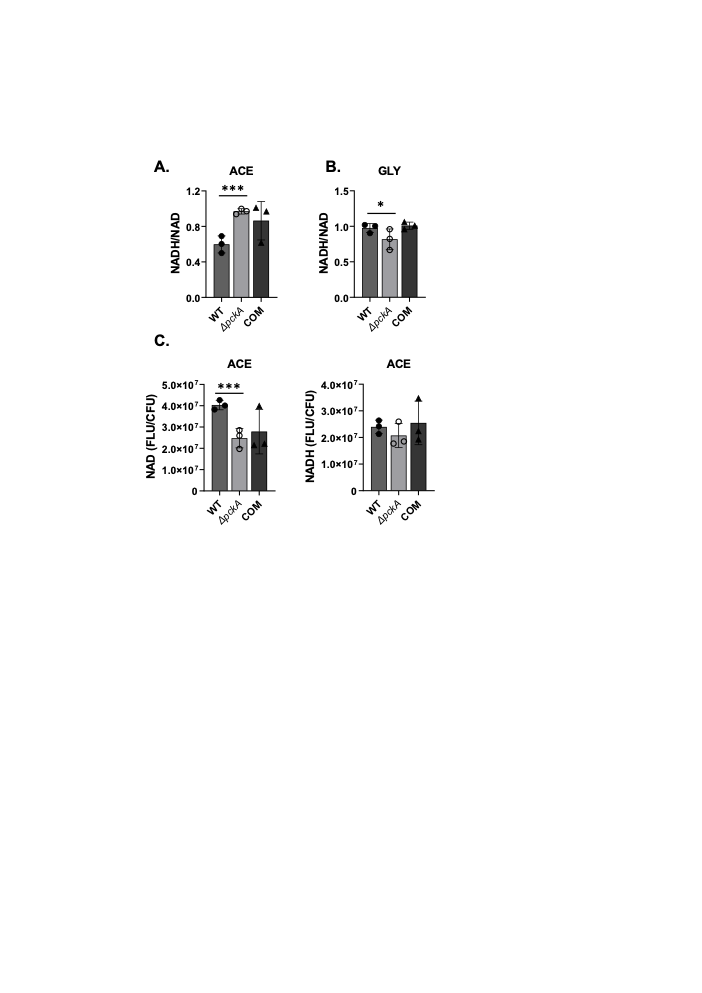

Supplement: FIG S4 [file mbio.03559-21-sf004.tif]

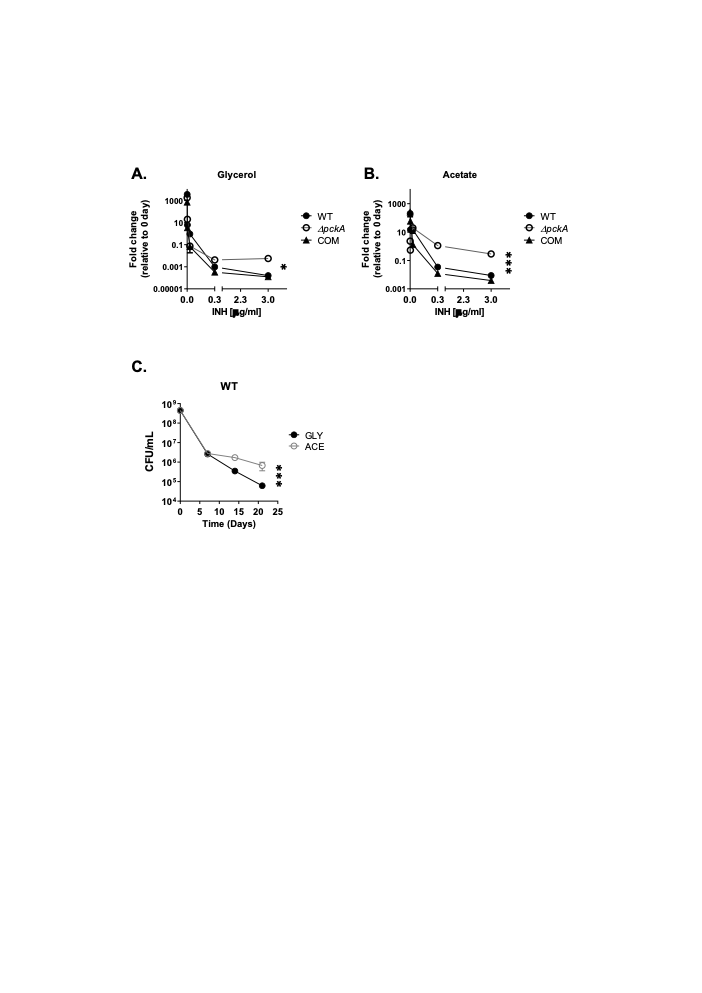

Supplement: FIG S5 [file mbio.03559-21-sf005.tif]

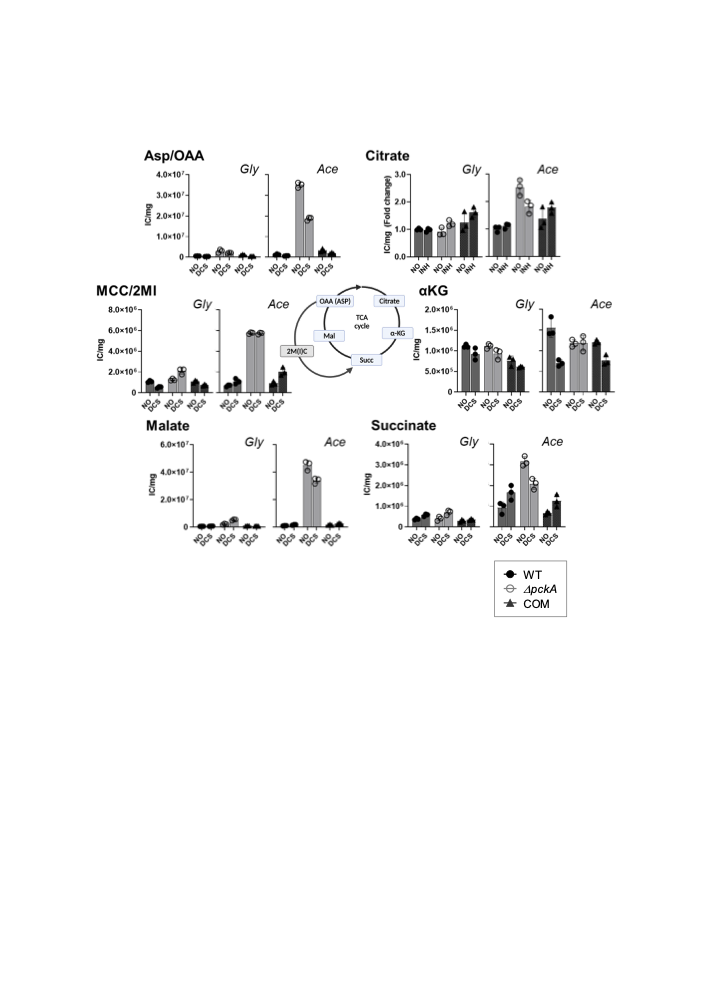

Supplement: FIG S6 [file mbio.03559-21-sf006.tif]

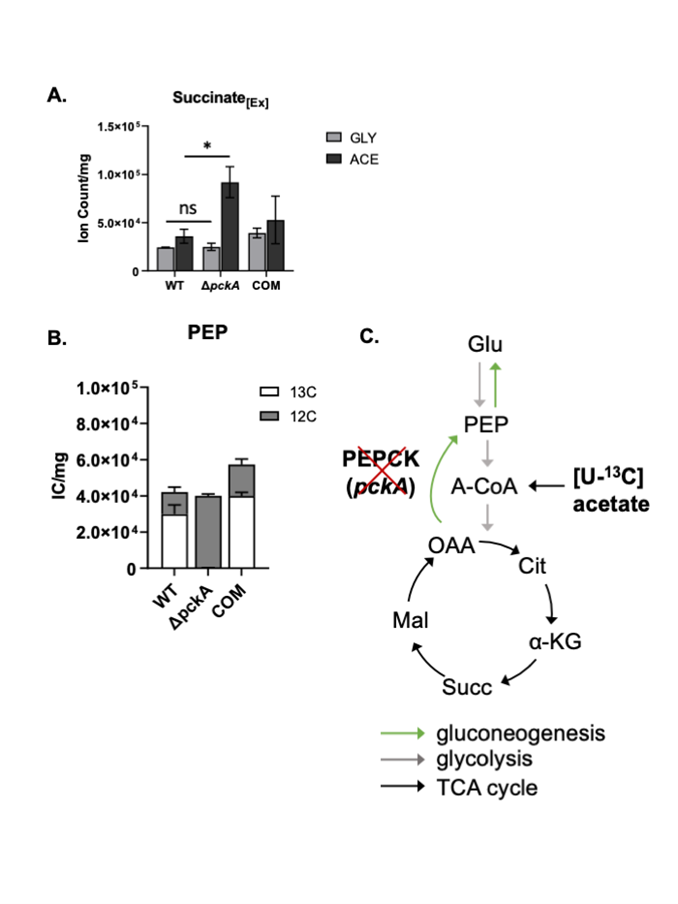

Supplement: FIG S7 [file mbio.03559-21-sf007.tif]

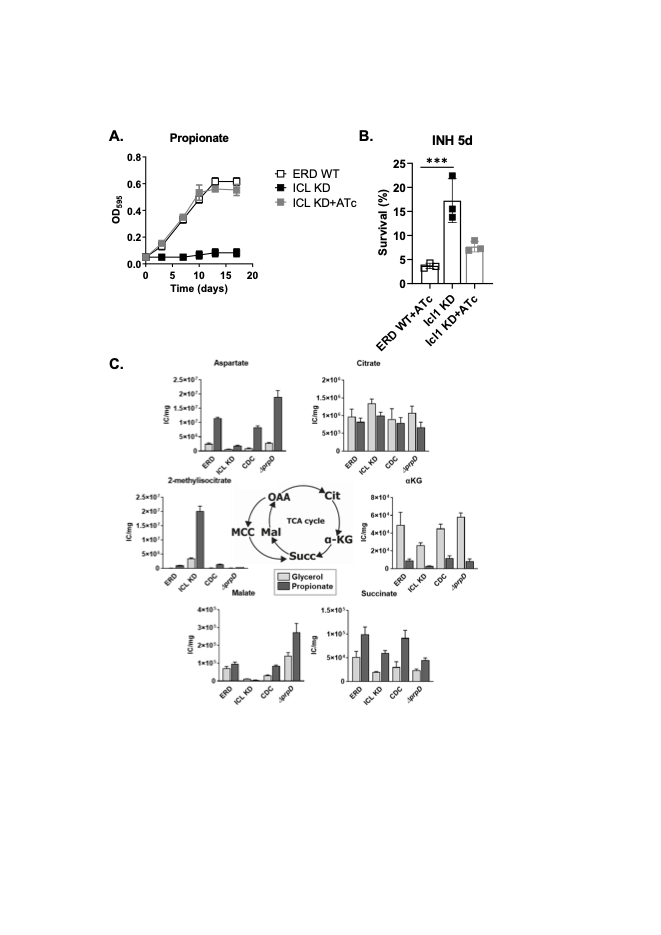

Supplement: FIG S8 [file mbio.03559-21-sf008.tif]
